# Use of "*biokit *HSV-2 Rapid Assay" to improve the positive predictive value of Focus HerpeSelect HSV-2 ELISA

**DOI:** 10.1186/1471-2334-5-84

**Published:** 2005-10-14

**Authors:** Rhoda Ashley Morrow, David Friedrich, Amalia Meier, Lawrence Corey

**Affiliations:** 1 Department of Laboratory Medicine, University of Washington, Seattle, Washington, USA; 2 Epidemiology, University of Washington, Seattle, Washington, USA; 3 Medicine, University of Washington, Seattle, Washington, USA; 4 Childrens Hospital and Regional Medical Center; Seattle, Washington, USA; 5 Fred Hutchinson Cancer Research Center; Seattle, Washington, USA

## Abstract

**Background:**

Commercially available assays to detect antibodies to the herpes simplex virus type 2 (HSV-2)-specific glycoprotein gG-2 have markedly improved serologic diagnosis of HSV-2 infection. However, even tests with high specificity can have low positive predictive values in low prevalence populations. HSV-2 is a chronic, life-long viral infection that requires both medical attention and potential alterations in health care strategy. As such, the concern for false positive diagnoses is high confirmatory testing is routine for other viral serologies such as HIV and hepatitis C. We evaluated such a strategy for HSV-2 serology by using an easily performed commercial test, *biokit*HSV-2 rapid test ("Biokit"; Biokit USA, Lexington MA) as a confirmatory test for the widely used gG-2 specific serology ("Focus;" HerpeSelect HSV-2 ELISA; Focus Diagnostics, Cypress CA).

**Methods:**

We tested 782 sera by Focus HSV-2 ELISA, Biokit, and the current gold standard test, Western blot (WB).

**Results:**

The positive predictive value of the Focus HSV-2 ELISA increased from 80.5% to 95.6% when Biokit testing was performed on sera that were initially positive by Focus HSV-2 ELISA. Confirmatory testing increased the specificity markedly among sera with Focus EIA values between 1.1 and 3.5: only 35% of low positive (index values 1.1–3.5) Focus HSV-2 ELISA results confirmed as positive by Biokit and WB compared with 92% of those with index values >3.5. Mathematical modeling of the data resulted in expected positive predictive values over 98% for populations with antibody prevalences typical of clinical practices in the US and Europe.

**Conclusion:**

Confirmatory Biokit testing of positive Focus HSV-2 ELISA results is fast, easy, and effective in reducing falsely positive HSV-2 antibody results. Patients, clinicians, and laboratories could benefit from the enhanced specificity of this simple HSV-2 serologic test combination.

## Background

Several studies over the last decade have shown the importance of subclinical HSV-2 reactivation in the epidemiology of HSV-2 infection. Over 95% of persons who are HSV-2 seropositive will reactivate and shed HSV-2 from genital sites and 70% of sexual and maternal-fetal transmission occurs from such subclinical shedding. As such, serologic detection of past HSV-2 increasingly is being recommended for a variety of immunocompetent and immunosuppressed populations. Several enzyme linked immunoassays for HSV-1 and HSV-2 antibodies to the type-specific glycoproteins, gG-1 and gG-2, respectively, are approved by the U.S. Food and Drug Administration. These methods are cost effective, widely available, and are the only commercial methods that accurately differentiate HSV-1 from HSV-2 antibodies. The HerpeSelect HSV-2 gG2 ELISA test (Focus Diagnostics) demonstrated a sensitivity of 96% in a group of pregnant women and 95% in an STD population of men and women [1]. Specificity of Focus HSV-2 ELISA also was high in these groups: 97 % in pregnant women and 96% in the STD population [1]. Both groups had relatively high HSV-2 seroprevalence by Western blot (WB); 25% of the pregnant women and 22% of the STD group had antibodies to HSV-2.

However, in select patient groups from several African countries, the Focus HSV-2 ELISA may give falsely positive results when compared with other gG-based tests such as the gG-2 monoclonal antibody inhibition assay [2] or WB [3,4]. A recent study of a low prevalence population suggests that falsely positive tests may not be limited to African populations [5]. All of these studies have found that Focus HSV-2 ELISA false positive results are far more likely with sera that have index values in the low positive range (1.1–3.5) than those that have index values above 3.5 [4,6]. As such, a confirmatory test to improve test specificity is desirable.

In 2000, a gG-2-based point of care membrane test, POCkit-HSV-2, was cleared by the US Food and Drug Administration for use with capillary blood and sera. This test showed high sensitivity and specificity in premarket trials against WB [7,8]. This test is now available as "*biokit*HSV-2 Rapid Test" from Biokit USA or as "SureVue-HSV-2" Rapid Test from Fisher HealthCare, Houston, TX. The *biokit*HSV-2 Rapid Test ("Biokit") is a readily accessible alternative to WB for confirmatory testing and can be performed easily on sera within a few minutes. Performing Biokit tests on sera that are initially positive by Focus HSV-2 ELISA could provide a useful strategy to increase the specificity of this HSV-2 serology.

To assess the value of biokit-HSV-2 as a confirmatory assay after an initial screening by Focus HSV-2 ELISA, we selected two sets of sera to study: 1) one from men at high risk for genital herpes and 2) one from an all-comer group of sera received by the University of Washington laboratory for HSV antibody testing. Biokit results were the same as WB results in 93.7% of these sera. Concordance of WB and Focus HSV-2 ELISA was 88.9%; concordance of Biokit and Focus HSV-2 ELISA was 86.7%. Using the Biokit result for sera positive by Focus HSV-2 ELISA increased the specificity from 93.2% to 98.7%. Positive predictive values increased from 80.5% for Focus HSV-2 ELISA to 95.6% when Biokit results were applied to sera that were positive by Focus HSV-2 ELISA.

## Methods

### Serology

Focus HerpeSelect HSV-2 ELISA ("Focus HSV-2 ELISA"; Focus Diagnostics, Cypress CA) was performed on each serum according to kit instructions. Sera with index values <0.9 were considered negative, those >3.5 as positive, values .9–1.1 (inclusive) were considered equivocal. Index values >1.1 to 3.5 were considered low positive.

The *biokit*HSV-2 Rapid Assay ("Biokit") Biokit USA, Lexington, MA) was performed according to kit instructions. Positive results were those in which the test spot was clearly colored red or pink. Negative results were those that had very faint or no color on the test spot. In a few cases, a colored ring appeared around an uncolored spot. These were scored negative.

The Western blot assay ("WB") for HSV-1 and HSV-2 was performed as described previously [9].

### Study subjects

Two groups were studied: 1) High-risk population: Through Dec 1, 2004, 1125 adult "men who have sex with men" (MSM) were tested by Focus HSV-2 ELISA to screen for enrollment into a National Institutes of Health trial evaluating acyclovir therapy to reduce acquisition of HIV-1 infection among HSV-2 seropositive persons (HIV Prevention Trial Network Protocol 039). As such, this serum subset was biased toward men who felt they had previously acquired HSV-2. Of the 388 sera found to be positive by Focus HSV-2 ELISA, 301 (77.6%) had sufficient volume to be used in the comparison study. Of the 718 that were seronegative by Focus HSV-2 ELISA, every 10^th ^sample with sufficient volume (N = 67) was selected to test by Biokit and WB. Sera that were indeterminate by Focus HSV-2 ELISA (N = 19) or WB (N = 14) were not tested by Biokit.

2) All-comer sera: We also tested 624 consecutive sera submitted for HSV WB testing to the University of Washington Virology Laboratory during a 4 week period in 2004. Of all subjects whose sera were received in 2004, 10% were under 20 years of age; 86% were 21–60 years old and 3% were over 60 years of age. Thus, some of our sample was likely to be pediatric. Of 148 sera that were positive by Focus HSV-2 ELISA, 141 were used in the comparison study. We selected 273 of the 469 samples that were negative by Focus HSV-2 ELISA for continued testing. Sera that were equivocal by Focus HSV-2 ELISA (N = 6) or by WB (N = 22) or by both tests (N = 1) were not analyzed further.

Samples from both groups were stripped of identifiers before performing the tests for this study. After de-identifying, each serum was stored at -20C and was thawed once for this study.

## Statistical measures

All Focus positive and low positive samples with sufficient volume were tested by WB and Biokit. Only a subset of Focus negative sera were run by WB or Biokit. Therefore, raw estimates of sensitivity, specificity and other predictive values were expected to be biased [10]. To adjust for this bias, we calculated expected confirmation rates among unconfirmed Focus negative results and incorporated these hypothetical results. Estimation was performed conservatively using binomial theory, first using positive rates for WB and Biokit in confirmed data to compute endpoints of a 95% confidence interval for the hypothetical number confirmed positive, then applying the extremes of this range to form two potential confidence intervals for each accuracy measure. A maximally-wide interval was built by combining these.

## Results

HSV-2 prevalence of the study populations was between 23.7 and 34.5% by Focus HSV-2 ELISA (Table 1). Overall, one-third of the HSV-2 seropositive sera had low positive results.

**Table 1:** HSV-2 status of study populations by Focus HSV-2 ELISA

Number of Sera (% of Group Total)						
	MSM Group		All-Comer Group		All Subjects	
HSV-2 Status (Index value)	Population	Sera Tested	Population	Sera Tested	Population	Sera Tested
Positive						
(All >1.1)	388 (34.5)	301	148 (23.7)	141	536	442
(Only >3.5)	245 (21.8)	194	110 (17.6)	106	355	300
(Only 1.1 – 3.5)	143 (12.7)	107	38 (6.1)	35	181	142
Equivocal (.9 – 1.1)	19 (1.7)	0	7 (1.1)	0	26	0
Negative (<0.9)	718 (63.8)	67	469 (75.2)	273	1187	340
Total	1125	368	624	414	1749	782

Because we were interested in evaluating the ability of the Biokit assay to serve as a confirmatory test for positive Focus HSV-2 ELISA samples, all seropositive sera with sufficient volume were used for subsequent WB and Biokit testing. In particular, 142 (78.5%) of the 181 low positive sera and 300 (84.5%) of the 355 high positive samples were tested by WB and Biokit. A subset of 340 (28.6%) of the 1187 seronegative sera by Focus HSV-2 ELISA was tested further (Table 1).

### Effect of index value on confirmation

**Figure 1:**
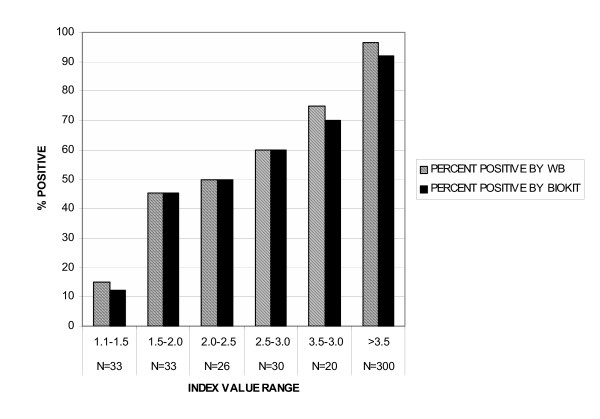
Western blot and Biokit results sorted by Focus HSV-2 ELISA index value. The proportion of sera that were positive by western blot (hatched bars) or by *biokit*HSV2 Rapid Test (solid bars) is shown for each range of index values obtained by Focus HerpeSelect HSV-2 ELISA. Numbers of sera (N) contributing to each subset are given below the designated index value ranges.

The proportion of sera that was positive by Focus HSV-2 ELISA and either WB or Biokit rose from <12–15% of sera with initial index values of 1.1–1.5 to >90% for index values >3.5 (Figure 1). The proportion of sera that confirmed at each index value level was very similar between Biokit and WB (Figure 1).

### Concordance among the 3 tests

**Figure 2:**
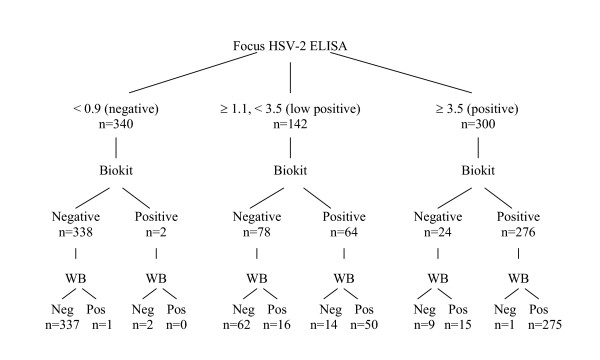
HSV-2 serology results by HerpeSelect HSV-2 ELISA ("Focus"), *biokit*HSV2 Rapid Test ("Biokit") and western blot (WB) in 782 sera.

The 3 tests had the same result in 662 (85%) of the 782 study sera (Figure 2) with 337 sera having concordantly negative results and 325 sera having concordantly positive results. Focus HSV-2 ELISA and WB were concordant in 339 negative sera and in 356 positive sera; overall 695 (88.9%). Focus HSV-2 ELISA and Biokit were concordant in 338 negative and 340 positive results; overall 678 (86.7%). WB and Biokit were concordant in 408 negative and 325 positive results for an overall concordance of 93.7%.

Of 300 sera that were positive by Focus HSV-2 ELISA with index values over 3.5, 275 (92%) confirmed by both Biokit and WB. Fifteen results confirmed as positive by WB, only (Figure 2). Low positive (index values 1.1–3.5) index values occurred in 142 sera. Only 50 (35.2%) confirmed as positive by both Biokit and WB while 62 (43.7%) confirmed by neither test (Figure 2). The low positive Focus HSV-2 ELISA index value group yielded the majority (N = 30) of the study's 49 discordant sera between Biokit and WB. Sera that were negative for HSV-2 antibody by Focus HSV-2 ELISA were nearly always negative by WB (339; 99.7%) or Biokit (338; 99.4%).

### Effect of Biokit confirmatory testing on test accuracy

To compute test accuracy, we applied data from the 340 sera that were seronegative by Focus HSV-2 ELISA to those Focus negative sera that were not run by WB or Biokit. We determined that approximately 2 (confidence interval [CI] 0–7) of the additional 847 seronegative sera would be positive by WB and about 5 (CI 0–12) would be positive by Biokit. Specificity of the Focus test was, in this way, estimated to be 93.2 (CI 91.8–94.6) with WB results as the gold standard. Specificity of the Biokit test, alone, against WB, was 98.4 (CI 97.5–99.3) (Table 2). Use of Biokit testing on all Focus HSV-2 ELISA positive sera improved estimated specificity from 93.2 to 98.7% without sacrificing sensitivity (99.1%). Positive predictive value improved from 80.5% to 95.6% and the negative predictive value remained at 99.7% (Table 2).

**Table 2:** Estimated sensitivity and specificity of HSV-2 tests

	Focus	Biokit	Focus Plus Biokit
Sensitivity	99.2 (96.3,100.0)	90.5 (86.1,94.0)	99.1 (96,100.0)
Specificity	93.2 (91.8,94.6)	98.4 (97.5,99.3)	98.7 (98.1,99.4)
Positive Predictive Value	80.5 (76.9,84.2)	94.5 (90.5,97.3)	95.6 (93.4,97.8)
Negative Predictive Value	99.7 (98.9,100.0)	97.5 (96.6,98.4)	99.7 (98.9,100.0)

Ranges indicated show the confidence intervals for accuracy; these also incorporate the uncertainty from having confirmed only a portion of Focus negatives. Biokit was used to confirm all sera that were initially positive for HSV-2 antibodies by Focus HSV-2 ELISA; the combined test results are shown in the "Focus Plus Biokit" column.

### Effect of HSV-1 antibody on discordance for HSV-2 between tests

Seventeen sera were false positive by Biokit as compared with WB. Two were also negative by Focus HSV-2 ELISA; 14 were low positive by Focus HSV-2 ELISA and 1 was positive by Focus HSV-2 ELISA with an index value >3.5. All 17 were positive for HSV-1 antibody by WB.

Of 86 sera with positive Focus test results but negative WB results, 68 (79.1%) were positive for HSV-1. The overall HSV-1 prevalence in the study was 64% (502 of 782) by WB. Thus, Biokit, and to a lesser extent, Focus HSV-2 ELISA specificity values appeared to be affected by the presence of HSV-1 antibodies. Conversely, WB could be falsely negative for HSV-2 in the presence of HSV-1 antibody.

### Predictive value of the test algorithm by population prevalence

We used the data for estimated sensitivity and specificity to evaluate the positive and negative predictive values that would be expected with populations that differ in prevalence of HSV-2 infection. As shown in Table 3, the use of the Biokit test to confirm initial Focus HSV-2 ELISA positive results (i.e. considering only results that are positive by both Focus HSV-2 ELISA and Biokit to be true positives) enhanced the positive predictive value (PPV) in all populations. PPV increased with increasing prevalence as predicted by Bayes theorem (PPV = Θp/ [Θp + (1-Φ)(1-p)], where p = prevalence, Θ = test sensitivity, and Φ = test specificity). For example, in groups with very low HSV-2 prevalence (10%) the PPV increased from 61.9 to 89.8%. For prevalences typical of antenatal practices (30%) and STD clinics (40–50%), the PPV of the test combination was over 97% and 98%, respectively.

**Table 3:** Positive and negative predictive values of HSV-2 test approaches by population prevalence

	Positive Predictive Value		
True Prevalence	Focus HSV-2 ELISA	Biokit	Focus HSV-2 ELISA+Biokit

			
10	61.9%	86.5%	89.8%
20	78.5%	93.5%	95.2%
30	86.3%	96.1%	97.1%
40	90.7%	97.5%	98.1%
50	93.6%	98.3%	98.8%
70	97.2%	99.3%	99.5%
90	99.2%	99.8%	99.9%
			
	Negative Predictive Value		
			
10	99.9%	98.9%	99.9%
20	99.8%	97.7%	99.8%
30	99.6%	96.0%	99.6%
40	99.4%	94.0%	99.4%
50	99.1%	91.2%	99.1%
70	98.0%	81.7%	97.9%
90	92.5%	53.6%	92.3%

Ranges indicated show the confidence intervals for accuracy; these also incorporate the uncertainty from having confirmed only a portion of Focus negatives. Biokit was used to confirm all sera that were initially positive for HSV-2 antibodies by Focus HSV-2 ELISA; the combined test results are shown in the "Focus Plus Biokit" column.

## Discussion

Commercially available type specific serologic testing for HSV-2 has markedly improved the ability to diagnose this common, widespread infection with significant clinical and therapeutic implications. While these assays have been extremely useful for research and epidemiological studies, there has been concern about their specificity for case management of HSV-2, especially in populations in which definitive data on seroprevalence are lacking [5]. Sera that have index values between 1.1 and 3.5 by Focus HSV-2 ELISA have the highest probability of being falsely positive. In our study, over one third of positive sera fell into this category of "low positive" index values and the majority of these sera did not confirm as positive by the Western blot assay (WB) [6].

One approach to this problem is to perform confirmatory assays on sera initially positive in the screening test. For example, the HerpeSelect immunblot assay is easy to run, relatively inexpensive at about $25 per test, and is FDA approved. However, the test is based on the same gG-2 antigen as the Focus HSV-2 ELISA and performs almost identically with that test [1]. As such, the immunoblot test has not been highly effective in discriminating falsely positive from truly positive Focus HSV-2 ELISA results [5]. Other confirmatory testing options require shipping sera to a reference laboratory for western blot (WB) or inhibition ELISAs that, while effective [6], can be time-consuming and expensive. We selected the *biokit *HSV-2 Rapid HSV-2 Test ("Biokit") as a confirmatory test because it is relatively inexpensive (about $20 per test), requires less than 10 minutes and no special equipment to perform, is FDA approved, and can be purchased and easily performed by any laboratory. The gG-2 antigen is a lectin-purified native protein as compared with the recombinant gG-2 used by Focus. Biokit's test also differs in presenting the antigen within a membrane while, in the Focus test, the gG-2 is sterically bound to a plastic microwell. These differences may result in different subpopulations of antibodies being detected so that a positive result in both tests is a more rigorous result than one provided by a single test.

Our study demonstrated that sera in the "low positive" index value range (1.1–3.5) by Focus HSV-2 ELISA had the highest proportion of discordant results among 3 tests: Focus, Biokit, and WB. Only 35.2% of low positive sera were positive by all 3 tests. In contrast, 99% of the Focus HSV-2 ELISA-negative sera were concordantly negative by all 3 tests and 92% of 300 sera with index values >3.5 were concordantly positive by all 3 tests. Low positive results are not rare. In the high risk MSM group in our study, 12.7% of sera, overall, and 36.9% of positive sera had index values of 1.1–3.5. In the all-comer group, 6.1% of sera, overall, and 25.7% of positive sera had low positive index values.

When sera with positive Focus HSV-2 ELISA results were run by Biokit, the combination provided a confirmed positive result in 340 (76.9%) of the 442 sera. By comparison, WB confirmed 356 (80.5%) of positive results. Moreover, 71 (16%) of the 442 positive sera were negative by both Biokit and WB; thus additional testing successfully identified false positive results in a significant proportion of the study population (4.1%). Overall, 89.6% of positive sera were given a higher quality answer as defined by confirming a positive result or by identifying a false positive. Either of these testing outcomes would serve to enhance the confidence of the laboratory providing results or the clinician preparing to counsel a patient.

The few false positive results from our testing strategy were all seen among those who were HSV-1 seropositive, a finding that has been previously described in a different patient population [5]. Several explanations are possible: First, the WB, which was the comparator assay, could be falsely negative; HSV-2 antibodies are more difficult to detect against a background of HSV-1 antibodies. Second, cross-reactive epitopes on glycoprotein G may affect the tests. While glycoprotein G molecules from HSV-1 and HSV-2 are predominantly distinct immunologically, there are regions of sequence homology and this effect cannot be definitively ruled out [11,12].

The two-step testing algorithm resulted in an increase in indeterminate results as defined by a positive Focus HSV-2 ELISA and a negative Biokit test. When Focus HSV-2 ELISA, alone, is used, 1% (26 of 1749) of study sera had index values in the indeterminate or "equivocal" range (0.9–1.1). When Biokit confirmatory testing was done, the additional unconfirmed results led to a total of 7.3% (128 of 1749) of samples with indeterminate outcomes. We feel this is a reasonable tradeoff for higher accuracy in determining HSV-2 infection status. Most of these additional indeterminate sera (N = 78) were low positive and most (N = 71) did not confirm by WB.

Several options are reasonable to determine HSV-2 serostatus in patients with Focus HSV-2 ELISA positive results that fail to confirm by Biokit. First, laboratories might consider retesting by Focus HSV-2 ELISA to rule out laboratory error. Reconstruction experiments have shown that low positive results can be artificially introduced by splashing or by dipping the pipette tip into a positive ELISA well, then into a negative well [4]. Seventy-four of the 86 sera that were positive by Focus HSV-2 ELISA but negative by WB were repeated and 11 sera (14.8%) retested as negative; all 11 were negative by Biokit. Thus, a repeat test can resolve the serostatus quickly, without redrawing blood from the patient, and, ideally, before the serology result leaves the laboratory. A second option is to re-test the patient in 6–12 weeks to rule out early seroconversion [13,14]. This option also applies to patients whose first sample was repeatedly low positive. A third option to establish HSV-2 serostatus is to send the sample to a reference laboratory for WB or to Focus Diagnostics for a gG-2 inhibition assay [6].

## Conclusion

Our study shows that Biokit confirmation of positive Focus results (index values >1.1) can substantially improve the positive predictive value of serologic screening for HSV-2 antibodies by Focus HSV-2 ELISA. Clinicians who counsel and manage patients with suspected herpes infections or asymptomatic, low-risk patients who wish to be screened for herpes may consider confirmatory testing to be both justified and cost-effective to increase accuracy. Laboratories now have available a quick and inexpensive means of providing a highly specific test combination for HSV-2.

## Competing interests

RAM has received speaking honoraria or consulting fees from Focus Technologies within the last 5 years. LC, AM, and DF report no competing interest. This study was supported by NIH grant AI 30731 and The Bill and Melinda Gates Foundation's Partners in Prevention Project Grant #26469; these entities do not have financial interest in the outcome of the study.

## Authors' contributions

RAM and LC designed the study and wrote the manuscript; DF established and maintained study data files and performed the basic data analyses. AM contributed to the study design and performed statistical analyses. All authors reviewed and approved the manuscript.

## Pre-publication history

The pre-publication history for this paper can be accessed here:


