# Correction: Use of "biokitHSV-2 Rapid Assay" to improve the positive predictive value of Focus HerpeSelect HSV-2 ELISA

**DOI:** 10.1186/1471-2334-7-11

**Published:** 2007-03-07

**Authors:** Rhoda Ashley Morrow, David Friedrich, Amalia Meier, Lawrence Corey

**Affiliations:** 1 Department of Laboratory Medicine, University of Washington, Seattle Washington, USA; 2 Epidemiology, University of Washington, Seattle, Washington, USA; 3 Medicine, University of Washington, Seattle, Washington, USA; 4 Childrens Hospital and Regional Medical Center, Seattle, Washington, USA; 5 Fred Hutchinson Cancer Research Center, Seattle, Washington, USA

## Abstract

As the competing interests for only the previous calendar year were included in the published article and it is journal policy to list competing interests for the previous five years, a full declaration of interests for the authors is now published.

As the competing interests for only the previous calendar year were included in the published article [1] and it is journal policy to list competing interests for the previous five years, a full declaration of interests for the authors is as follows:

During this time, Dr. Corey was a consultant to a company involved in developing an HSV vaccine (Antigenics). His role was to advise the company on what potential HSV proteins they should select for their peptide based vaccine.

Dr. Corey has been the lead investigator of several trials that used HSV serologies as a critical component of the study. These trials have used either the University of Washington Western blot or the Focus assay followed by the University of Washington Western blot. All grant funds associated with these trials were paid to the University of Washington to support personnel and supplies associated with the work. Dr. Corey received no salary support from these funds.

Dr. Corey is associated with a company which sponsors an informational website that is available to anyone with internet access without charge [2]. The website was created as a public service to health care providers and the lay public. Dr. Corey writes content appearing on the website. In the years 2002 to 2004, Focus Technologies, as well as several private individuals and companies involved in HSV therapy provided funding to support development of new content as well as periodically revise and maintain the website. Dr. Corey received compensation for writing and revising content on the website which provides information on the epidemiology, clinical manifestations, therapy and diagnosis of HSV infection. His editorial responsibilities did not involve the area of HSV serology.

## Pre-publication history

The pre-publication history for this paper can be accessed here:


